# Enhancement of muscle activation during squat exercise: evaluation with magnetic resonance imaging

**DOI:** 10.1007/s00421-025-05856-5

**Published:** 2025-07-01

**Authors:** Riccardo G. Sorrentino, Andrej Vovk, Dušan Šuput, Leonidas G. Ioannou, Veronika Mekjavic, Rodrigo Fernandez-Gonzalo, Matej Supej, Igor B. Mekjavic

**Affiliations:** 1 Department of Automatics, Biocybernetics and Robotics, Jozef Stefan Institute, Ljubljana, Slovenia; 2 Jozef Stefan International Postgraduate School, Jamova Cesta 39, 1000 Ljubljana, Slovenia; 3 Faculty of Medicine, University of Ljubljana, Ljubljana, Slovenia; 4 Department of Laboratory Medicine, Division of Clinical Physiology, Karolinska Institutet, Stockholm, Sweden; 5 Unit of Clinical Physiology, Karolinska University Hospital, Stockholm, Sweden; 6 Faculty of Physical Education, University of Ljubljana, Ljubljana, Slovenia

**Keywords:** Whole-body vibration, Magnetic resonance imaging, Resistance exercise, Muscular activation, Muscle activity

## Abstract

We evaluated whether vibration augmented muscle use during squat exercise with magnetic resonance imaging, with two methods: spin–spin relaxation time (T2 mapping), and volume analysis.

Male participants (*n* = 13) visited the facility on two occasions. During the first visit, each participant completed a resistance exercise (RE) comprising four sets of 12 repetitions of triple extension squats. Before and immediately after completion of the exercise, magnetic resonance imaging of their thigh and calf muscles was performed. During the second visit, participants performed resistance vibration exercise (RVE) using the same protocol, but standing on a vibration plate that provided a whole-body vibration stimulus (20 Hz, 3.5 mm amplitude) during the exercise.

RE promoted increased T2 in *vastus lateralis*, *vastus medialis* and *vastus intermedius* muscles (*p* < 0.0001). The same activation was observed for RVE, with no differences compared to RE. The volumetric method was less effective in assessing muscle activation. Only the *vastus medialis* and *vastus intermedius* muscle volumes were significantly greater post-exercise compared to pre-exercise for both RE (both muscles: *p* = 0.0009) and RVE (respectively: *p* = 0.005 and *p* = 0.009). No muscular activation was detected for calf either with T2 or volumetric method.

Resistance exercise increased T2 and volume of some muscles of the thigh. The addition of whole-body vibration to resistance exercise does not enhance T2 relaxation time or volume accumulation.

## Introduction

Resistance exercise is considered the gold-standard for improving muscle structure and function (Aube et al. 2022; Shaw et al. 2009). Enhancement of muscle activation by traditional exercises, such as squats, deadlifts and their variations, are well documented (Schoenfeld et al. 2016). Performing consistent resistance exercise training, and consequently increasing muscle activation, promotes both neural and hormonal neuromuscular adaptations, concomitant with muscle hypertrophy (Mendes Ritti Dias et al. 2005; Paulsen et al. 2003), which eventually results in increased strength and muscle size (DeFreitas et al. 2011; Del Balso and Cafarelli 2007; Folland and Williams 2007).

A variety of novel exercise modalities aimed at enhancing muscular training have been introduced and investigated, ranging from electrical stimulation (Doucet et al. 2012), blood flow restriction training (Lorenz et al. 2021) and whole-body vibration (Rittweger 2010). The latter consists of applying mechanical vibrations through a vibrating platform to the whole body, both with (Delecluse et al. 2003) and without resistance exercise (Tankisheva et al. 2013).

Evidence suggests that the stimuli generated by vibrations might increase neuromuscular activation, thus leading to enhanced muscle recruitment and potentially enhanced training adaptation (Hazell et al. 2010; Marín et al. 2015; Marín and Cochrane 2021). However, the additional benefit of combining vibration with resistance exercise remains equivocal (Arora et al. 2021; Artero et al. 2012; Celik et al. 2022). Methodological discrepancies, including variations in vibration type (vertical or rotational), intensity (frequencies and amplitudes), duration of exposure and population investigated, contribute to the lack of consensus in the literature, precluding definitive conclusions.

One of the main questions regarding whole-body vibration (WBV) is its effects on muscular activation. Several studies have investigated muscle activation following WBV protocol with electromyography (Giminiani et al. 2013, 2015; Lienhard 2014; Lienhard et al. 2015; Lim 2003). Concerns regarding EMG efficacy arise due to potential vibration interference with signal detection, addressable via bandpass filters (Pollock et al. 2010) and also it is possible that the acute muscular activation is a reflex triggered by α and γ motor neurons which modulate muscle stiffness (Cardinale and Bosco 2003; Celik et al. 2022; Rittweger 2020).

Magnetic resonance imaging (MRI) is widely regarded as a reliable technique for investigating muscle structure due to its ability to produce high-resolution images of muscle tissue. This non-invasive method offers detailed insights into muscle structure (Murphy et al. 1986), activation patterns and alterations in response to exercise or other stimuli (Cagnie et al. 2011; Dickx et al. 2010; Fernandez-Gonzalo et al. 2016; Hooijmans et al. 2020). Notably, MRI can detect increases in the spin–spin T2 relaxation time of water within muscle tissue, which serves as an indicator of muscle activity. The nuclear magnetic resonance signal arises from the magnetic behaviour of hydrogen nuclei in tissue water and fat. When exposed to a strong magnetic field, these nuclei are excited by a resonant radio frequency pulse, causing synchronized oscillation and generating a detectable signal. Once the pulse stops, the nuclei gradually lose phase coherence, leading to signal decay, known as T2 relaxation. This spin–spin relaxation is independent of magnetic field strength and increases after muscle activity. In MRI, T2 relaxation time reflects fluid shifts, intracellular acidification, and muscle recruitment, providing insight into metabolic and physiological changes (Cagnie et al. 2011; Dickx et al. 2010; Kinugasa et al. 2006; Patten et al. 2003; Shaikh et al. 2023). It is generally thought that exercise promotes the accumulation of osmolytes (i.e., phosphates, lactate, sodium) in the cytoplasm and an influx of free-water in the active muscles which increase the volume of intracellular space. This results in a prolonged T2 following exercise, which is used as an indirect marker of muscle recruitment and workload (Cagnie et al. 2011; Dickx et al. 2010; Price et al. 2003). The T2 effect is often still detectable 20–30 min after the exercise, hence osmotic water uptake solely due to lactate accumulation is insufficient to account for overall water uptake. In fact, the mechanisms which leads to water retention after muscle use are not fully understood. Along with T2 analysis, assessing muscle volume is crucial for understanding structural adaptation in response to exercise. Changes in volume can provide insights about muscle use (Wilcox et al. 2021). Measuring both volume and T2 relaxation time provides a comprehensive view of both functional and morphological effects of acute exercise on muscles.

According to Fisher et al. (1990), the increase in T2 can be quantitatively associated with muscle activity. Unlike electromyography (EMG), which is limited to surface muscle activity, MRI can assess the activity of deeper muscle structures without the need for invasive procedures. However, the use of MRI as an exercise evaluation tool is challenging due to the sometimes limited availability of MRI facilities, the need for highly trained technicians, and the high costs associated with its use. Few studies discussed this methodology and the possibility to measure muscle activation following exercise, conceivably, due to the aforementioned difficulties in performing this analysis.

The aims of this study were threefold: (i) utilize MRI to quantify muscle use and acute volumetric changes in all the muscles in the thigh and calf (both anterior and posterior sections) following exercise; (ii) compare muscle activation, as reflected by T2 relaxation times, between conventional resistance exercises and whole-body vibration resistance exercise; and (iii) assess the inter-operator reliability of these measurements to establish the robustness of MRI-based evaluations in this context.

## Methods

### Participants

Healthy male participants (*n* = 13) aged 24 ± 4 (mean ± standard deviation) years, with a mass of 76.9 ± 7.6 kg and height of 180.4 ± 3.4 cm, participated in the study. The sample size was determined based on findings from previous studies (Kinugasa et al. 2006; Tawara 2022). All participants met the inclusion criteria, which included: age between 18 and 40 years, being physically active, and the ability to perform squat exercises in accordance with established guidelines. During a preliminary visit to the laboratory, prior to the experimental trials, participants' squat technique was assessed by two strength and conditioning experts. In addition, participants were familiarized with the exercise that they had to perform either with and without vibration. All participants were familiar with resistance exercise and, aside from one participant, were engaged in regular resistance training. The study protocol was approved by the University of Ljubljana, Faculty of Sports’ Committee for Ethical Issues in the Field of Sport (Reference number: 033-10/2023-2). All participants provided their written informed consent to participate in the study, which was performed according to the guidelines of the Declaration of Helsinki, excluding clause 35 (i.e., the study was not registered in a publicly accessible database).

### Experimental protocol

The subjects were requested to visit the MRI facility on two occasions separated by 2 weeks. To limit measurement error, MRI scans, interaction with participants and exercise supervision, were performed by the same operators and MRI technicians.

Upon arrival at the facility, participants completed a medical questionnaire to survey factors that might interfere with MRI scans, and underwent a consultation with medical personnel. Subsequently, participants changed into shorts, and MRI scans were conducted on two segments of the right leg: one positioned 10 cm below the patella to assess the calf muscles  and another 10 cm above the patella to evaluate the thigh muscles. A trained technician assisted participants in positioning themselves within the MRI scanner. The scanner was situated in an adjacent room, separated from the control room by a glass partition, enabling personnel to monitor participants and provide assistance in case of emergency. A communication system facilitated interaction between the MRI personnel and subject. The duration of the MRI scan was approximately 15 min. Following the scan, participants performed resistance exercise (RE) comprising four sets of twelve repetitions of body weight triple extension squats, with one minute of rest between sets and exercises. The exercise was paced using a metronome, with a cadence of 3 s for the descending phase, 3 s for the ascending phase, and a 1-s isometric contraction at the end of the triple extension squat, specifically when participants were on their toes. This exercise protocol was chosen based on its intensity and regimen employed in a previous study (Mekjavic et al. 2023). The exercise was performed under the supervision of a strength and conditioning expert. Following the exercise session, participants were asked to dry themselves and promptly re-entered the MRI scanner for a subsequent scan.

During the second visit, which took place 2 weeks after the first, the procedure was similar to that of the first visit, with the exception that the participants performed the resistance exercise on a rotational vibration platform, thus conducting resistance vibration exercise (RVE). Vibrations were delivered using a Galileo rotational platform (Novotech, Germany), operating at a frequency of 20 Hz. The amplitude, which varied based on each participant's foot placement on the platform, averaged around 3.5 mm. The vibration intensity was selected according to WBV frequencies known to induce muscle activation (14–40 Hz), while also ensuring participant safety and enabling exercise without excessive foot displacement during vibration.

### Muscle magnetic resonance imaging

#### MRI sequences

Magnetic resonance imaging (MRI) data were acquired on a 3.0 T Philips Achieva TX scanner (Philips Healthcare, Best, NL) with a 12-channel coil. The imaging protocol consisted of T2-weighted turbo spin echo sequences with the following parameters: repetition time (TR) = 2000 ms, echo time (TE) = 13 ms (first echo) with six additional echoes acquired with an echo spacing (ΔTE) of 13 ms (last TE = 78 ms), acquisition matrix = 560 × 560, flip angle = 90°, voxel size = 0.286 × 0.286 × 2.2 mm^3^, number of slices = 10, and SENSE acceleration factor = 2.5.

### Data analysis

Data were recorded through the MRI acquisition software. Muscle segmentation was performed with semi-automatic muscle segmentation software using deep registration-based label propagation.^1^

**Fig. 1:**
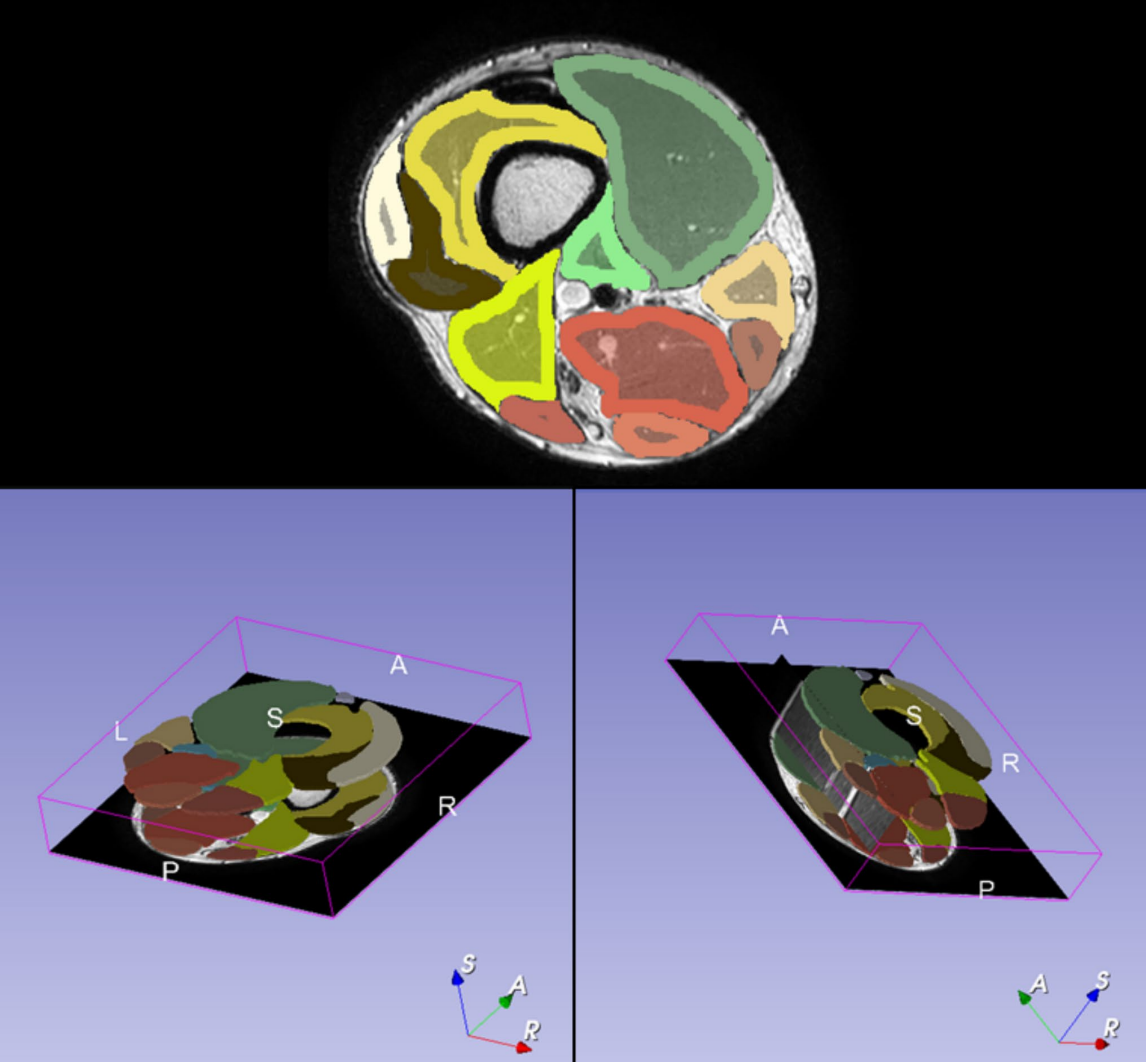
Visual representation of volumetric analysis of muscles. The image in the upper panel is an example of the axial view of the thigh scan. The images in the lower panel illustrate the three-dimensional depiction of the segmentations performed on the first (slice 1) and the last slice (slice 14) of the MRI scans. The volume (cm^3^) between the two segmentations was recreated by calculating the area between individual muscle segmentation. The model was created and modified by using *Slicer ver. 5.6.2* (https://www.slicer.org/). Scan panel legend: L = left, R = right, A = anterior, P = posterior. Bottom right axes legend: S = sagittal, A = axial, R = coronal

This segmentation method relies on registration-based label propagation, which provides 3D muscle delineations from a limited number of annotated 2D slices (Fig. 1). ITK Snap software (Open-source: http://www.itksnap.org version 4.0) was used to delineate axial view of muscles on top and bottom slices. Fat tissue and major vessels were not included in the segmentation. From the segmented volumes ROI statistics were obtained using AFNI’s tool 3dROIstats (Cox 1996). To isolate the water signal and minimize fat interference in T2 mapping, data were analysed using a biexponential T2 fitting model, accounting for distinct relaxation rates of water and fat. T2 maps were calculated with MyoQMRI open-source software available at https://github.com/fsantini/MyoQMRI and introduced in previously published method (Santini et al. 2021).

The calf and thigh muscle segmentation was performed by three independent evaluators. All performed the segmentation according to the same guidelines, namely taking care to exclude fat and bone tissue, and blood vessels.

Data regarding T2 mapping and volume were then stored in Excel files as comma separated values. Volumetric values were converted from voxels to cm^3^ and used for the analysis. To evaluate the consistency of measurements between operators, the Sørensen–Dice index or DICE coefficient was calculated for muscle segmentation performed by three operators. The DICE coefficient was derived using the formula (Sørensen–Dice index):* DICE* = *2* ×*|A ∩ B| / (|A|* +*|B|)*; with A and B are two data samples. A DICE score of 1 signifies perfect agreement between the measurements, whereas a score of 0 denotes no agreement. The DICE score was calculated for every muscle of the thigh and calf. To detect potential outliers, considering the intervariability in participants' responses to exercise and the baseline body composition, Z-score analysis was applied to all datasets. Data points with Z-scores exceeding −2 or +2 were identified as outliers and consequently excluded from further analysis.

Data were checked for normal distribution with a Shapiro–Wilk test. For the analysis, only identifiable muscles were included; muscles that were consistently unobservable were excluded. Total muscular activation within the thigh and calf was calculated by summing the volumes and T2 values of all respective muscles.

Data were  investigated using paired multiple *t*-tests with the Holm–Bonferroni correction method for multiple comparisons. When the normality distribution of data was not met, Multiple Wilcoxon tests with correction for multiple comparisons was used. When a difference was observed in a muscle, a mixed effect model with *exercise* (RE and RVE) and *time* (PRE and POST) was used to evaluate whether the exercise had an effect. Given the two-week interval between visits, post hoc analyses with Šidák correction for multiple comparisons and effect size estimation were conducted within the model to evaluate potential differences between baseline measurements.

Analysis of variance (2 way-ANOVA) can determine whether there is a statistically significant difference between two measures; however, it does not provide information about the actual size or practical significance of the observed difference. To address this limitation, this study also employed effect size analysis to quantify the magnitude of the difference. Due to the relatively small sample size (less than 20), Hedge’s G was used as it includes a correction factor appropriate for small samples (Sullivan and Feinn 2012).

The statistical analysis and graphical representation were performed with GraphPad Prism version 10 (GraphPad Software Inc., Dotmatics, Boston), while effect sizes, volumetric conversion and *Z*-scores were performed with Microsoft^tm^ Excel.

## Results

The DICE coefficient revealed a very high measurement-agreement between operators. Some small muscles were not always visible in all participants, thus in those muscles the DICE coefficient was lower compared to all other muscles. For the whole-thigh (the average DICE score among all muscles) a score of 0.91 ± 0.08 was achieved, while for the calf it was 0.88 ± 0.07. The highest DICE coefficient recorded for thigh muscles was 0.98 for the *vastus medialis,* while the lowest was 0.52 for the *adductor magnus.* For calf muscles the highest DICE coefficient was 0.98 for the *gastrocnemius lateralis*, while the lowest was 0.50 for the *extensor digitorum longus.* Analysis of variance indicated no significant baseline differences between RE and RVE for either volumetric measurements or T2 values. Nevertheless, small to moderate effect sizes were observed for the volumetric analysis in the *vastus medialis* (*G* = 0.49), *vastus intermedius* (*G* = 0.44), and *vastus lateralis* (*G* = 0.26), whereas no notable effect sizes were found between baselines T2.

### Effect of resistance exercise

#### Calf volume and T2 mapping

**Fig. 2:**
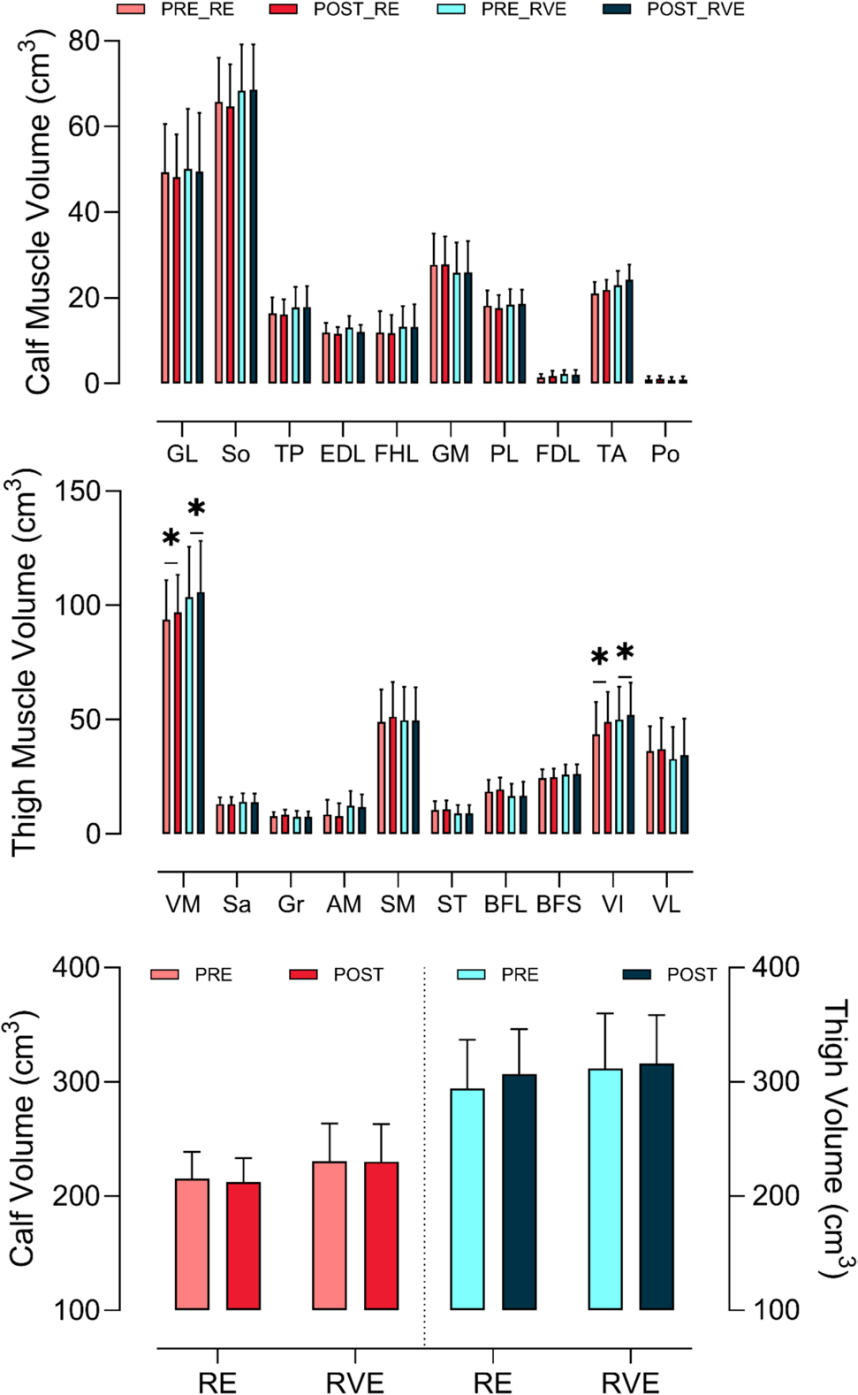
The mean (SD) volume of calf (top panel), and thigh muscles (middle panel) before (pre) and after (post) resistance exercise without (RE) and with (RVE) vibration. The bottom panel presents the mean (SD) volume (cm^3^) of all calf and thigh muscles before and after RE and RVE. Asterisk (*) indicates the main effect of time for both RE and RVE. Top panel legend: GL = Gastrocnemius Lateralis, So = Soleus, TP = Tibialis Posterior, EDL = Extensor Digitorum Longus, FHL = Flexor Hallucis Longus, GM = Gastrocnemius Medialis, PL = Peroneus Longus, FDL = Flexor Digitorum Longus, TA = Tibialis Anterior, Po = Popliteus. Middle panel legend: VM = Vastus Medialis, Sa = Sartorius, Gr = Gracilis, AM = Adductor Magnus, SM = Semimembranosus, ST = Semitendinosus, BFL = Bicep Femoris Long, BFS = Bicep Femoris Short, VI = Vastus Intermedius, VL = Vastus Lateralis

**Fig. 3:**
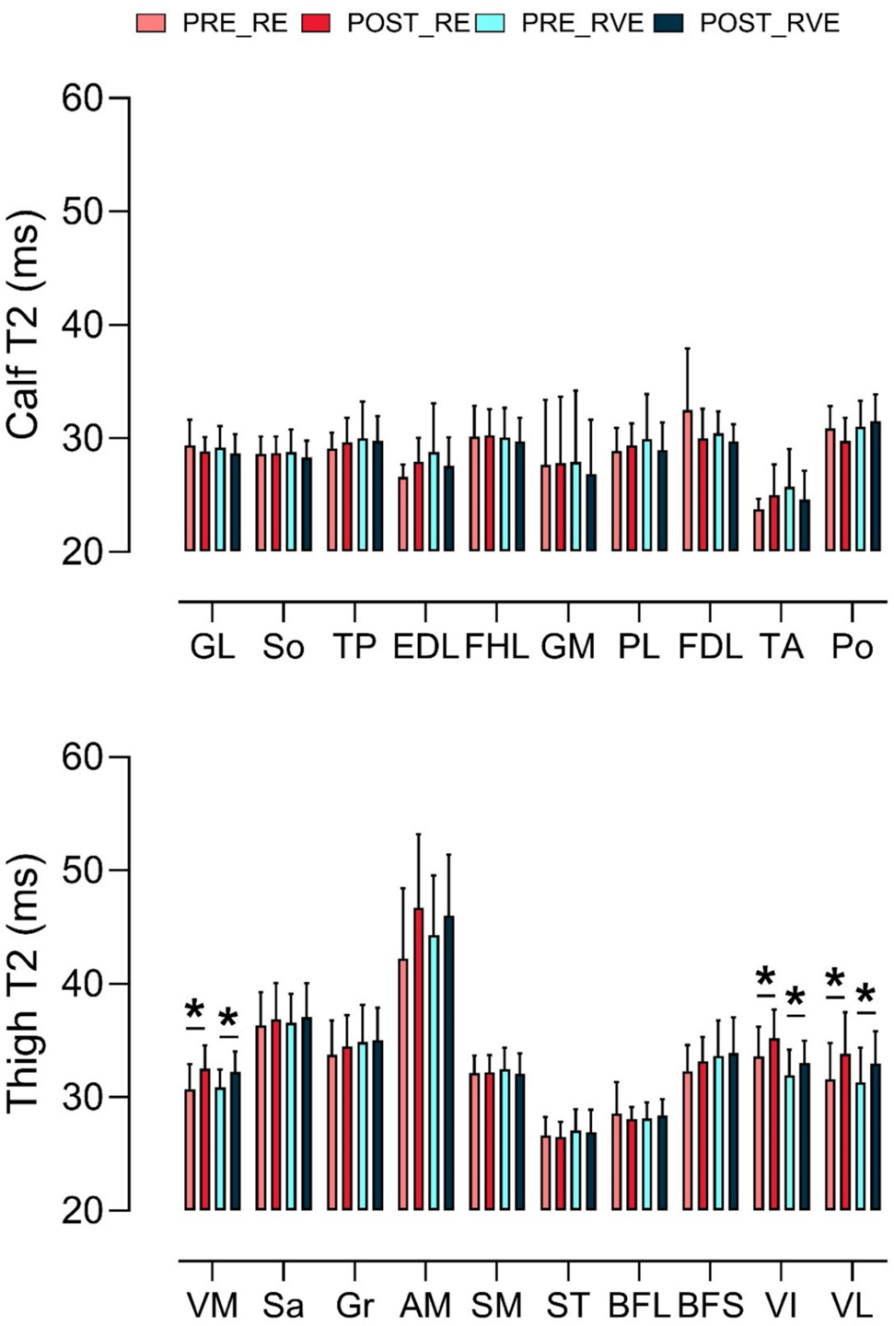
The mean (SD) T2 (ms) results for the calf (upper panel) and thigh (middle panel) muscles, before (pre) and after (post) resistance exercise without (RE) and with (RVE) vibration exercise. Asterisk (*) indicates the main effect of time for both RE and RVE. Legend: GL = Gastrocnemius Lateralis, So = Soleus, TP = Tibialis Posterior, EDL = Extensor Digitorum Longus, FHL = Flexor Hallucis Longus, GM = Gastrocnemius Medialis, PL = Peroneus Longus, FDL = Flexor Digitorum Longus, TA = Tibialis Anterior, Po = Popliteus. Middle panel legend: VM = Vastus Medialis, Sa = Sartorius, Gr = Gracilis, AM = Adductor Magnus, SM = Semimembranosus, ST = Semitendinosus, BFL = Bicep Femoris Long, BFS = Bicep Femoris Short, VI = Vastus Intermedius, VL = Vastus Lateralis

Resistance exercise with or without whole-body vibration did not promote any muscular activation or changes in muscle use either for single calf muscles and for all calf muscles analysed collectively. Delta analysis revealed no differences between interventions as shown in Figs. 2 and 3.

#### Thigh volume and T2 mapping

A *time* effect was recorded for acute volume data in *vastus medialis* and *vastus intermedius* (*p* < 0.0001), indicating that both resistance exercise with and without vibration increased the volume of these muscles, without differences across exercise regimens. Following resistance exercise (RE), the *vastus medialis* volume increased by 4% (from 93.84 ± 17.17 to 96.97 ± 16.46 cm^3^), while the *vastus intermedius* volume increased by 11% (from 43.73 ± 14.03 to 48.99 ± 13.10 cm^3^).

**Fig. 4:**
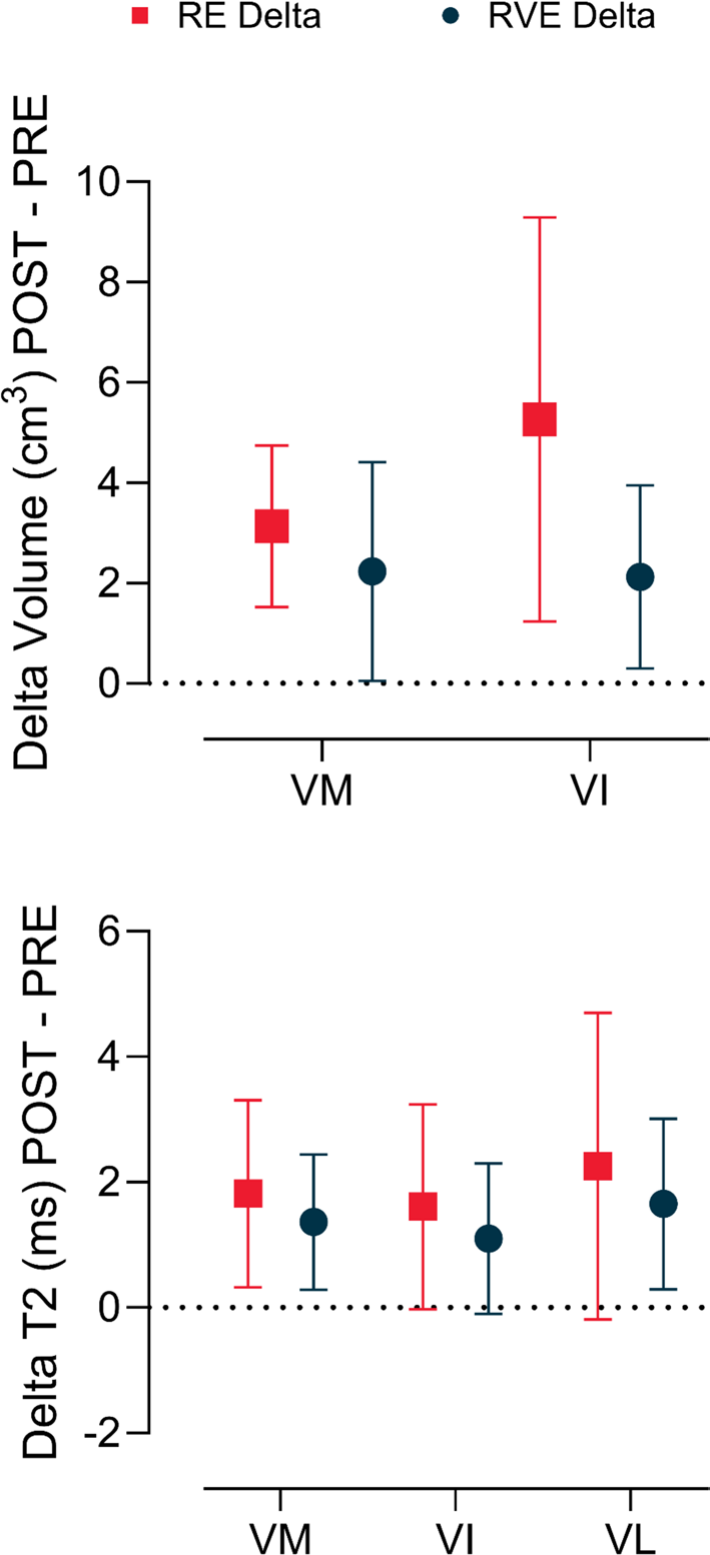
Upper panel: mean (SD) differences in muscle volume (cm^3^) of the vastus medialis (VM) and vastus intermedius (VI) muscles observed after (post) and before (pre) resistance exercise without (RE, red squares) and with (RVE, blue circles) vibration. Lower panel: mean (SD) differences in T2 results of the vastus medialis (VM), vastus intermedius (VI) and vastus lateralis (VL) muscles observed after (post) and before (pre) resistance exercise without (RE, red squares) and with (RVE, blue circles) vibration

A similar response was observed following resistive vibration exercise (RVE), with *vastus medialis* volume increasing by 3% (from 103.69 ± 21.96 to 105.92 ± 22.30 cm^3^) and *vastus intermedius* volume increasing by 5% (from 50.09 ± 14.43 to 52.22 ± 13.96 cm^3^). Additionally, a significant time effect was detected (*p* = 0.0001) for whole thigh volume, indicating increases in both RE (from 294.12 ± 42.68 to 306.83 ± 39.45 cm^3^) and RVE (from 311.39 ± 48.38 to 316.38 ± 42.12 cm^3^), with no significant differences between exercise modalities. PRE vs POST effect size analysis revealed no practical difference for the abovementioned muscles. Delta analysis on PRE and POST of *vastus medialis* and *vastus intermedius* showed no difference between RE and RVE (Fig. 4).

A significant time effect was observed for T2 mapping in the *vastus medialis, vastus intermedius* and *vastus lateralis* (*p* < 0.0001). Following RE, T2 relaxation time increased by 6% in both the *vastus medialis* (from 30.72 ± 2.23 to 32.53 ± 2.02 ms) and *vastus intermedius* (from 33.59 ± 2.62 to 35.20 ± 2.51 ms). Additionally, a 7% increase in T2 time was recorded for the *vastus lateralis* (from 31.53 ± 3.24 to 33.82 ± 3.68 ms).

Following RVE, T2 relaxation time increased by 5% in the *vastus medialis* (from 30.87 ± 1.57 to 32.24 ± 1.81 ms), 4% in the *vastus intermedius* (from 31.95 ± 2.24 to 33.05 ± 1.94 ms), and 6% in the *vastus lateralis* (from 31.33 ± 3.07 to 32.98 ± 2.85 ms). No significant differences were detected between exercise regimens, indicating a comparable T2 relaxation time response across conditions. Moderate (*G* > 0.50) and large effect sizes (*G* > 0.80) between PRE and POST were recorded for *vastus medialis, vastus intermedius and vastus lateralis* in both groups. Delta analysis on PRE and POST of *vastus intermedius, vastus lateralis* and *vastus medialis* showed no differences between RE and RVE (Fig. 4).

## Discussion

The main findings of this study confirm that MRI is a reliable tool to obtain acute muscular use data and that during the squat resistance exercise (RE) the addition of whole-body vibration (RVE) does not increase muscle use assessed by water and metabolites accumulation through muscle functional MRI (mfMRI). In addition, the DICE score revealed that data regarding muscle segmentation among operators were overlapping, hence indicating a high reliability between measurements. These results should be interpreted in light of the non-randomized sequence of RE and RVE, which constitutes a potential methodological limitation. Additionally, the effect sizes reported for certain muscles at baseline in the volumetric analysis may reflect residual influences from untracked activity between timepoints and possible carry-over effect on volumes.

Muscle activation is commonly assessed using electromyography (EMG), but it has limitations. Surface EMG is restricted to superficial muscles and is affected by skin impedance, adipose tissue, and electrode placement, increasing measurement variability. Assessing deeper muscles requires needle EMG, which is invasive and may cause discomfort, particularly during dynamic exercise. For these reasons, mfMRI has been proposed as a complementary method to indirectly assess muscle use activation trough the measurement of water and metabolites accumulation (Akima et al. 2000; Cagnie et al. 2011; Fleckenstein et al. 1988).

Previous results confirm that measuring T2 relaxation time using MRI is a reliable method providing insights about muscle use (Cagnie et al. 2011; Dickx et al. 2010; Price et al. 2003; Shaikh et al. 2023), in addition, there is evidence of the linear association between mfMRI and EMG analysis (Adams et al. 1992; Dickx et al. 2010; Yue et al. 1994). The pioneering work of Tesch (1993) provided an in-depth analysis of muscle activation after several lifting exercises. This study validated the hypothesis posited by Fleckenstein et al. (1988) demonstrating that MRI enables the direct visualization of all muscles engaged, as well as those not engaged, during a specific movement, thereby offering insights into the primary musculature involved in a given exercise. In a recent study, Tawara (2022) demonstrated that T2 relaxation time using MRI can detect muscle activation even following a mild body weight exercise. The main limitations in using fMRI to detect muscular activation is the need to perform the examination immediately after exercise, given that the effects of exercise on water and metabolite accumulation tend to decay rapidly (Fisher et al. 1990). Therefore, in our study the exercise was performed in a room adjacent to the MRI device in order to start the MRI scans within just a couple of minutes (< 2 min) after the exercise.

The effects of superimposed vibration on muscles are highly debated in the literature. Rosenberg and colleagues  (2014) found that, after signal filtering, higher EMG signals were found in the rectus femoris during squats with 20 Hz of superimposed vibration. Pollock et al.  (2010) reported similar results. There is evidence of an  increased ATP consumption (indirectly indicating muscle use) after superimposed vibration at 20 Hz (Zange et al. 2009), supported also by increased respiratory O_2_ consumption due to vibration with exercise (Cochrane et al. 2008). These results are supported also by training studies (Delecluse et al. 2003; Karatrantou et al. 2013; Petit et al. 2010; Rubio-Arias et al. 2018) which suggest that the increased performance after exercise with vibration cannot be attributed to a placebo effect. On the contrary, other studies provide evidence of no additional benefits from vibration training (Artero et al. 2012; Celik et al. 2022; de Ruiter et al. 2002, 2003). Indeed, one study reported that resistive squatting elicited greater leg press force performance when performed without vibration (Kvorning et al. 2006). The discrepancies in the literature may be attributed to many factors influencing the results of both acute and long-term interventions. The type of exercise, such as multijoint movements, and its modalities, such as isometric and concentric actions, can significantly influence the effects of whole-body vibration (WBV) on muscles (Cardinale and Wakeling 2005).

The significant increase in T2 in the *vastus medialis*, *vastus intermedius* and *vastus lateralis* is consistent with the primary muscles engaged during a squat exercise, as these muscles are integral components of the quadriceps and are in line with the findings of Tesch (1993). The lack of an increase in T2 time in the calf muscles aligns with previous findings in the literature. Alkner and Tesch (2004) have demonstrated, with similar exercise, that this muscle group is particularly challenging to effectively target. The non-uniform changes between and within muscles observed in this study have been already discussed previously (Kubota et al. 2007; Tesch 1993; Yue et al. 1994). It is known that during exercise, not all muscles involved in the movement experience the same level of fatigue. Some muscles may be more activated than others, and different regions of the same muscle respond non-uniformly to the effort (Kubota et al. 2007; Mendez-Villanueva et al. 2016; Yue et al. 1994).

The findings of this study would support previous literature suggesting no vibration effects on muscle activation (Arora et al. 2021; Celik et al. 2022; de Ruiter et al. 2002, 2003). T2 relaxation time indirectly reflects the accumulation of water and metabolites, leading to muscle oedema. However, this process is highly variable, and the precise physiological mechanisms underlying water and metabolite retention in active muscles remain unclear. One potential explanation for these discrepancies lies in the physiological response to vibration-induced muscle activation. Vibration is thought to elicit passive muscle contractions through mechanoreceptors, specifically the muscle spindles, which detect vibratory stimuli. This triggers a stretch-reflex response transmitted to the α-motoneurons via monosynaptic (I-afferents) or polysynaptic (II-afferents) pathways, ultimately activating muscle spindles and facilitating contraction (Desmedt and Godaux 1978; Granit et al. 1956; Rittweger 2010; Ritzmann et al. 2010). Another potential explanation is the intensity of vibrations. While the intensity used in this study may have been sufficient to enhance neuromuscular activation, as observed in previous research (Rosenberger et al. 2014; Zange et al. 2009), it may not have been adequate to induce significant metabolite accumulation and water retention. The discrepancy between increased neuromuscular activation via monosynaptic pathways and the absence of water and metabolite accumulation following superimposed vibration in muscles, warrants further investigation. Combining electromyography (EMG) and T2 mapping may provide deeper insights into the physiological mechanisms underlying enhanced muscle activation and tissue fluid dynamics.

As expected, muscle volume did not exhibit significant changes following a single exercise session. While volumetric analysis can reflect functional effects, as muscle swelling typically occurs after intense exercise, it primarily captures structural adaptations that become more evident after repeated training sessions. Since higher exercise intensity correlates with increased T2 values, a similar trend may occur in volumetric changes over time. Additionally, the bodyweight exercise performed in this study may not have been sufficient to induce substantial muscle swelling or detectable volume alterations.

The results from the current study should be interpreted with some considerations in mind. This study employed a body weight exercise, which was reportedly sufficient to detect muscle activation with MRI (Tawara 2022). The exercise was not sufficiently intense for activating the calf muscles, which are known to require higher loads to increase in size and performance due to their constant activation in countering gravity while standing. Future investigations, might employ different training loads to investigate load effect on the deeper musculature detected with the above-mentioned MRI techniques. In addition, future research should further explore the effects of vibration on acute muscle function by integrating T2 MRI and electromyography (EMG) assessments as well as employing different vibration intensity.

## Conclusion

Muscle use can be assessed using volumetric and T2 MRI analysis; however, T2 relaxation time may provide a more precise representation of acute muscle utilization when imaging is performed immediately post-exercise. The application of superimposed vibration did not result in increased muscle use assessed by water or metabolite accumulation through T2 relaxation-time analysis.

## Data Availability

The raw data will be made available by the authors upon request.

## Footnotes

1 https://hal.science/hal-03945559v1* available at: *https://github.com/nathandecaux/labelprop
